# Uncovering the invisible—prevalence, characteristics, and radiomics feature–based detection of visually undetectable intraprostatic tumor lesions in ^68^GaPSMA-11 PET images of patients with primary prostate cancer

**DOI:** 10.1007/s00259-020-05111-3

**Published:** 2020-11-18

**Authors:** Constantinos Zamboglou, Alisa S. Bettermann, Christian Gratzke, Michael Mix, Juri Ruf, Selina Kiefer, Cordula A. Jilg, Matthias Benndorf, Simon Spohn, Thomas F. Fassbender, Peter Bronsert, Mengxia Chen, Hongqian Guo, Feng Wang, Xuefeng Qiu, Anca-Ligia Grosu

**Affiliations:** 1 Department of Radiation Oncology, Medical Center – University of Freiburg, Faculty of Medicine, University of Freiburg, Robert-Koch Straße 3, 79106 Freiburg, Germany; 2 German Cancer Consortium (DKTK), Partner Site Freiburg, Freiburg, Germany; 3 Department of Urology, Medical Center – University of Freiburg, Faculty of Medicine, University of Freiburg, Freiburg, Germany; 4 Department of Nuclear Medicine, Medical Center – University of Freiburg, Faculty of Medicine, University of Freiburg, Freiburg, Germany; 5 Institute for Surgical Pathology, Medical Center – University of Freiburg, Faculty of Medicine, University of Freiburg, Freiburg, Germany; 6 Department of Radiology, Medical Center – University of Freiburg, Faculty of Medicine, University of Freiburg, Freiburg, Germany; 7 Department of Urology, Affiliated Drum Tower Hospital, Medical School of Nanjing University, Nanjing, China; 8 Department of Nuclear Medicine, Affiliated Drum Tower Hospital, Medical School of Nanjing University, Nanjing, China

**Keywords:** Prostate cancer, PSMA-PET, Intraprostatic lesions, Multifocality, Radiomics

## Abstract

**Introduction:**

Primary prostate cancer (PCa) can be visualized on prostate-specific membrane antigen positron emission tomography (PSMA-PET) with high accuracy. However, intraprostatic lesions may be missed by visual PSMA-PET interpretation. In this work, we quantified and characterized the intraprostatic lesions which have been missed by visual PSMA-PET image interpretation. In addition, we investigated whether PSMA-PET-derived radiomics features (RFs) could detect these lesions.

**Methodology:**

This study consists of two cohorts of primary PCa patients: a prospective training cohort (*n* = 20) and an external validation cohort (*n* = 52). All patients underwent ^68^Ga-PSMA-11 PET/CT and histology sections were obtained after surgery. PCa lesions missed by visual PET image interpretation were counted and their International Society of Urological Pathology score (ISUP) was obtained. Finally, 154 RFs were derived from the PET images and the discriminative power to differentiate between prostates with or without visually undetectable lesions was assessed and areas under the receiver-operating curve (ROC-AUC) as well as sensitivities/specificities were calculated.

**Results:**

In the training cohort, visual PET image interpretation missed 134 tumor lesions in 60% (12/20) of the patients, and of these patients, 75% had clinically significant (ISUP > 1) PCa. The median diameter of the missed lesions was 2.2 mm (range: 1–6). Standard clinical parameters like the NCCN risk group were equally distributed between patients with and without visually missed lesions (*p* < 0.05). Two RFs (local binary pattern (LBP) size-zone non-uniformality normalized and LBP small-area emphasis) were found to perform excellently in visually unknown PCa detection (Mann-Whitney *U*: *p* < 0.01, ROC-AUC: ≥ 0.93). In the validation cohort, PCa was missed in 50% (26/52) of the patients and 77% of these patients possessed clinically significant PCa. The sensitivities of both RFs in the validation cohort were ≥ 0.8.

**Conclusion:**

Visual PSMA-PET image interpretation may miss small but clinically significant PCa in a relevant number of patients and RFs can be implemented to uncover them. This could be used for guiding personalized treatments.

**Supplementary Information:**

The online version contains supplementary material available at 10.1007/s00259-020-05111-3.

## Introduction

Current advances in prostate cancer (PCa) imaging, the transition towards precision medicine and tailored treatment, have led to the evolvement of focal therapy as treatment alternatives for localized PCa [1]. Various treatment options like radiotherapy (RT), high-intensity focal ultrasound, or focal cryotherapy have been proposed to apply (i) a tumor-directed treatment by targeting solely the visible intraprostatic tumor volume while sparing the rest of the prostatic gland [2] or by (ii) intensifying the treatment on the intraprostatic tumor volume while treating the rest of the prostatic gland conventionally [3].

Multiparametric magnetic resonance imaging (mpMRI) is the standard of care (SOC) for the initial staging of patients with primary PCa [1] and is momentarily utilized for targeted biopsy concepts [4] and for focal therapy strategies [5, 6] among other indications. However, mpMRI can underestimate the true tumor mass [7–9]. The prostate-specific membrane antigen (PSMA) is selectively overexpressed in most PCa cells and can be traced by radiolabeled peptide ligands ^68^Ga-PSMA-11 in positron emission tomography (PET) [10]. PSMA-PET/CT imaging has already been established as the gold standard for restaging in recurrent PCa after curative treatment [11, 12]. Furthermore, there is growing evidence that PSMA-PET/CT is a suitable replacement for conventional imaging in the primary setting, providing superior accuracy at initial staging [13, 14]. Considering intraprostatic tumor detection, previous studies suggested that PSMA-PET outperformed MRI in the detection of intraprostatic tumors with high sensitivities of 64–89% [7, 15, 16]. However, PCa is renowned for its multifocality. Mouarviev and colleagues analyzed 947 prostatectomy specimens and only 22% of PCa were unifocal whereas 78% were multifocal with 2.24 lesions on average [17]. For choline-PET, Souvatzoglou et al. [18] proved that a significant amount of intraprostatic lesions might be missed by visual PET image interpretation due to small lesion size or lesion configuration. Currently, the characteristics of the visually undetectable lesions in PSMA-PET are unknown.

Obviously, the accurate detection of the entire clinically significant tumor mass is mandatory for a safe implementation of focal therapy strategies in patients with primary PCa. With the rise of big data analysis, the computer-based extraction of quantitative imaging parameters called radiomics features (RFs) may enable new concepts for personalized medicine. PET-derived RFs may be used as biomarkers to predict treatment outcomes and to characterize tumor biology non-invasively [19]. For example, RFs from ^68^Ga-PSMA-11 PET images provided excellent results in Gleason score discrimination [20].

The first aim of this study was to quantify and to characterize the clinically significant PCa lesions which have been missed by visual ^68^Ga-PSMA-11 PET image interpretation. Secondly, we analyzed whether basic clinical parameters and ^68^Ga-PSMA-11 PET-derived RFs can detect and characterize these visually undetectable intraprostatic lesions and their underlying multifocality. PCa distribution in co-registered whole-mount sections served as the reference in internal training and external validation cohorts.

## Methodology

### Patient cohorts

The study consisted of two cohorts including patients with low-, intermediate-, and high-risk PCa according to the National Comprehensive Cancer Network (NCCN) classification (NCCN.org). Inclusion criteria were defined as conduction of ^68^Ga-PSMA-11 PET/CT scans at initial staging; and available histology reference material: whole-mount surgery specimen. Patients with any therapy interventions or ongoing androgen deprivation therapy before PET scans were excluded. A detailed description of the two patient cohorts is given in Table 1. A cohort of 20 prospectively enrolled patients (Freiburg, Germany) was utilized for training. The external validation cohort comprised 52 retrospectively enrolled patients from Nanjing, China. The study was approved by each local ethics committee (Ethics committee University of Freiburg: 469/14; Ethics committee Nanjing University: 2017-147-01) and written informed consent was obtained from all patients.

**Table 1:** Patient characteristics

	Training cohort	External validation cohort
Center	Freiburg, Germany	Nanjing, China
Enrollment	Prospective	Retrospective
Histology	Whole-mount histopathology	Whole-mount histopathology
Patients (*n*)	20	52
Age in years (median, range)	67 (48–76)	69 (55–84)
ISUP (*n*)		
1	1	6
2	7	13
3	5	16
4	4	8
5	3	9
pT stage (*n*)		
2	8	17
3a	6	24
3b	6	11
4	0	0
Median iPSA (ng/ml)	16.9 (5.6–218)	13.5 (4.1–110)

The patient characteristics of the different cohorts as well as the study design and histologic reference material are listed as an overview. Whole-mount histopathology is generated by radical prostatectomy

*iPSA* initial PSA

### ^68^Ga-PSMA-11 PET imaging

#### Freiburg

Details of our radiolabeling protocol of ^68^Ga-PSMA-11 can be found in Zamboglou et al. [21]. All patients had to fast for at least 4 h before the administration of the radiopharmaceutical (median activity: 206 MBq, range: 114–251 MBq) and had to void before starting the PET scan. One hour after intravenous injection, patients underwent the whole-body PET scan. PET/CT whole-body acquisition protocols were performed on three cross-calibrated different systems from Philips (The Netherlands): 10 patients in GEMINI TF TOF 64 (TF64), 7 in GEMINI TF 16 Big Bore (BB), and three patients in Vereos (V). All systems fulfilled the requirements of the European Association of Nuclear Medicine (EANM) ^18^F-imaging guidelines and obtained EANM Research Ltd. (EARL) accreditation. A contrast-enhanced diagnostic CT (120 kVp, 100–400 mAs, dose modulation) or a low-dose CT (120 kVp, 25 mAs) was performed. Based on the corresponding CT dataset, PET images were corrected for scattering and attenuation. Please see our previous work [20] for the reconstruction methods for all scanners. All systems resulted in PET images with a voxel size of 2 × 2 × 2 mm. Images were normalized to decay corrected injected activity per kg body weight (SUV [g/ml]). The acquisition time per bed position ranged from 2 to 3 min and the overlap in the axial field of view (FOV) was of 53% for GEMINI systems and 43% for V.

#### Nanjing

As described previously, ^68^Ga-PSMA-11 was synthesized with an ITG semi-automated module (Germany, Munich) [22]. One hour before scanning, all patients had an intravenous injection with ^68^Ga-PSMA-11 (median, 131.72 MBq, range 130.6–177.6 MBq). All PET-CT scans were performed in a uMI 780 PET-CT scanner (United Imaging Healthcare (UIH), Shanghai, China). A CT scan (130 keV, 80 mAs) and a static emission scan, corrected for dead time, scatter and decay, were acquired from the vertex to the proximal legs. The voxel size of the PET images was 2.3 × 2.3 × 2.7 mm. All images were normalized to decay corrected injected activity per kg body weight (SUV [g/ml]). The images were corrected by CT-based attenuation correction and then iteratively reconstructed by HYPER UVT, composed of point spread function model and TOF information to realize the high-definition reconstruction, further reducing the line of response width as well as noise and distortion.

#### PET image processing

**Fig. 1:**
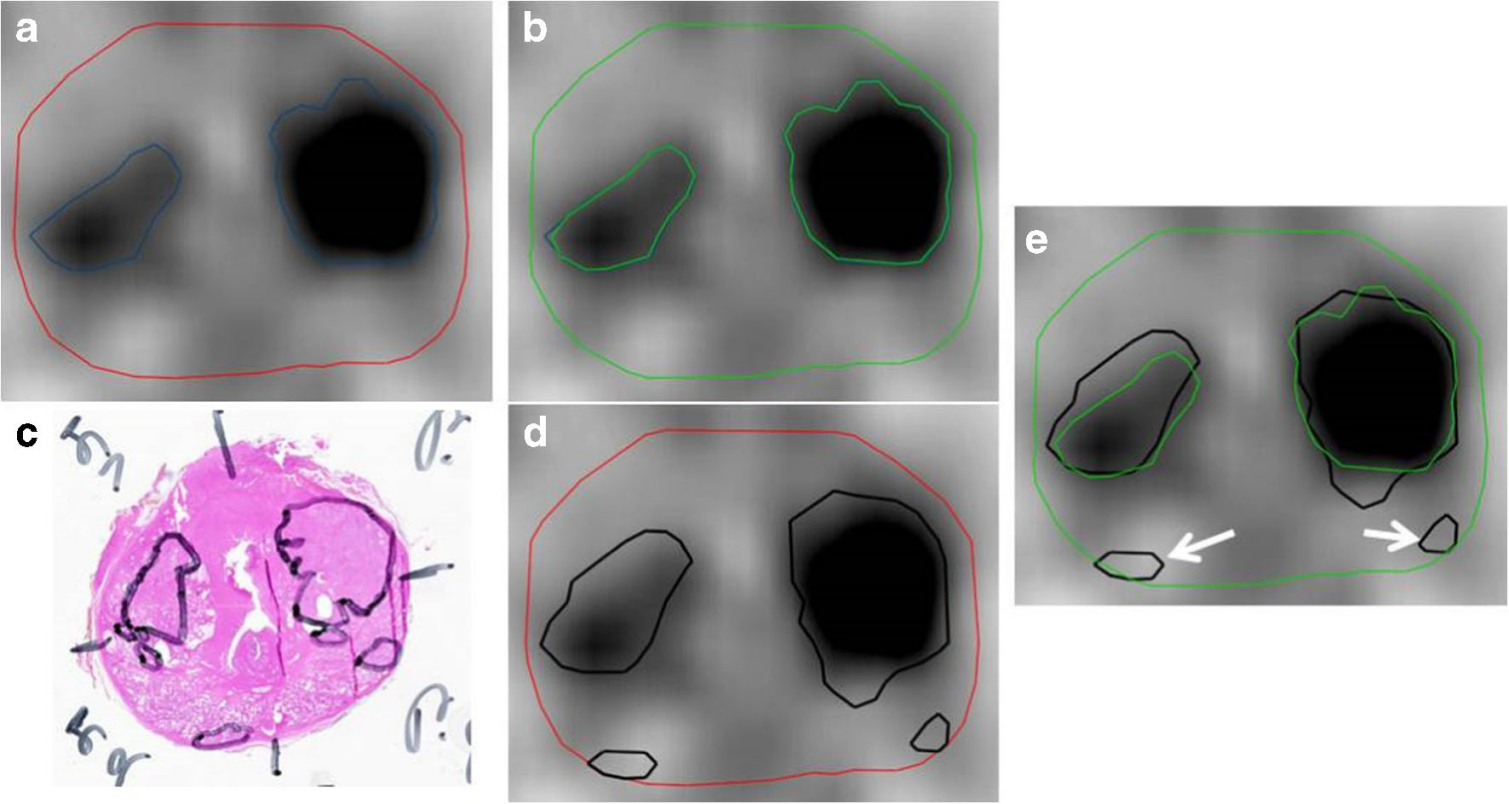
Gross tumor volume in axial ^68^Ga-PSMA-11 PET images (GTV-PET, blue) was delineated by two readers in consensus (**a**). The prostatic gland was segmented by reference to the co-registered CT images (red). The GTV-PET was subtracted from the prostatic gland to create non-PCa-PET (green) (**b**). **c** H&E-stained whole-mount section with marked PCa in black. Whole-mount slices were co-registered with PET images and in **d**, the PCa is projected on the axial PET slice. Finally, the presence of PCa lesions in non-PCa-PET was obtained from each patient on the entire prostate level and half prostate level (right vs. left lobe). **e** The representative patient had PCa lesions (white arrows) in non-PCa-PET in both lobes, respectively. Both lesions had a maximum diameter of 5 mm and a lenticular shape. Both lesions were ISUP 2/Gleason core 7a (3 + 4)

Two readers (AB and CZ) delineated the intraprostatic gross tumor volume (GTV-PET) in ^68^Ga-PSMA-11 PET images of all patients in consensus using a validated contouring technique [23]. PET images were scaled from 0 SUV to 5 SUV and any signal higher than the adjacent background in more than one image slice was interpreted as PCa. The prostatic gland volume was segmented by CZ under consideration of the corresponding CT scan. GTV-PET was subtracted from the prostatic gland volume to obtain a 3D volume defined as non-PCa tissue in ^68^Ga-PSMA-11 PET (non-PCa-PET, Fig. 1). The median volume of non-PCa-PET was 25.7 ml (range: 7–93.7). In the training cohort, non-PCa-PET volumes were additionally divided into left and right lobes in order to perform half-gland analyses. In the validation cohort, RF extraction was performed with and without previous resampling (nearest-neighbor interpolation to obtain isotropic 2 × 2 × 2 mm voxels).

### PET images/histology correlation

#### Training cohort, Freiburg

The information of the 3D distribution of PCa in the radical prostatectomy specimen had been obtained using a published, in-house co-registration protocol [24]. After formalin fixation, the resected prostate underwent an ex vivo CT scan in a customized localizer. To ensure equal cutting angles of the ex vivo CT slices and tissue specimen, whole-mount step sections were cut every 4 mm using an in-house cutting device. Subsequently, tissue specimens were paraffin-embedded and sliced. Staining with hematoxylin and eosin was performed via routine protocols and one of two experienced pathologists segmented PCa tissue and classified the PCa lesions according to ISUP grade. Histology slices were registered on the corresponding ex vivo CT images under consideration of intraprostatic and extraprostatic markers. Based on the PCa segmentations on the co-registered histopathology slides, the PCa contour was manually contoured on each corresponding ex vivo CT image. Subsequently, the PCa contours were interpolated by 2-mm expansion in both *Z*-axis directions to create a model of the 3D distribution of PCa. Considering the non-linear transformations of the prostatic gland after resection, ex vivo CT (including histology information) was manually registered on in vivo CT (from ^68^Ga-PSMA-11 PET/CT scans) utilizing non-rigid deformations. The prostatic border and intraprostatic landmarks (e.g., calcifications) served as reference points. The alignment of in vivo CT and PET scan was based on hardware co-registration of the scanners. A misalignment between PET and in vivo CT images occurred (up to 2 cm) in 4 of the 20 patients and a manual adaption was performed with rigid registration.

#### Validation cohort, Nanjing

After radical prostatectomy, whole-mount tissue was fixed in 10% formalin. The tissue was paraffin-embedded, cut into slices of up to 5–6 mm by a microtome, and stained with hematoxylin-eosin. All whole-mount histology slides were digitalized by a scanning system (NanoZoomer Digital Pathology, Shizuoka, Japan). Subsequently, all pathologic images were interpreted by a dedicated genitourinary pathologist. PCa lesions were identified and segmented. The corresponding ISUP scores were assigned.

Clinically significant PCa was defined accordingly to Bonekamp et al. [25] as any lesion with International Society of Urological Pathology (ISUP) score of > 1 or Gleason score of > 6 (3 + 3) [26] for both cohorts.

### Radiomics feature extraction

The RFs were computed using the open-source python package PyRadiomics version 2.02 [27] which incorporates most of the definitions of the image biomarker standardization initiative [28]. As described in our previous work [20], feature reduction was performed upfront by a phantom study to discard RF with significant inter-scanner variability comparing the three different PET/CT scanner systems in Freiburg (BB, TF, V). The NEMA NU2 phantom was employed and 9 contours of variable size and intensity were segmented. In each patient, 154 RFs which were proofed robustly were extracted per volume (entire non-PCa-PET, left lobe, and right lobe non-PCa-PET). RFs were derived from a 3D volume [29] and SUV values of the voxels within the contour were discretized with a fixed bin width (*W* = 0.05) [20], resulting in different numbers of bins depending on the range of SUV values in the contour. First-order features and texture features, using gray-level voxel values and spatial information, were derived. Texture features were calculated on four different matrices: gray-level co-occurrence matrix (GLCM [30]), the gray-level run length matrix (GLRLM [20]), the gray-level size-zone matrix (GLSZM [31]), and the neighborhood gray-tone difference matrix (NGTDM [32]). In addition to RF extraction from the original image, we applied a local binary pattern (LBP) filter [33]. This filter is described to detect microstructures by locally comparing voxel gray levels and creating a binary pattern code [34]. Further details on the feature extraction settings and the experimental setup are given in Supplementary Material 1.

### Statistical analysis

Statistical analyses were performed in R software v.3.6.1 and GraphPad Prism v.8.1 (GraphPad Software, USA). ^68^Ga-PSMA-11 PET-derived RFs were summarized using descriptive statistics. To compare two different groups of metric variables, the two-tailed Mann-Whitney *U* test for non-pairwise testing and Fisher’s exact test were used for comparison of categorical variables, respectively. This was because most of the variables (> 80%) were not normally distributed in the Shapiro-Wilk normality test. The *p* values were adjusted for multiple testing by controlling the false discovery rate with Benjamini and Hochberg’s method [35] and *p* < 0.05 was considered to be statistically significant. Correlation analyses were performed by calculating the Spearman correlation coefficient (*ρ*) and the area under the receiver-operating characteristic curve (AUC-ROC) was evaluated. The latter analyses were carried out in whole-gland and half-gland levels due to two reasons: (i) to prove whether the RFs are robust when being extracted in two different volumes of interest and (ii) under consideration of therapeutic consequences. A RF extraction on whole-gland level enables ultra-focal therapy approaches (treating only the visible tumor mass) whereas RF extraction on half-gland level enables hemi-gland therapy (treating only the tumor-affected side).

Considering several drawbacks in PET/histopathology registration in the validation cohort (please see “Discussion” for further details), RF extraction in this cohort was only performed in patients with histologically confirmed and clearly autonomous lesions in non-PC-PET.

## Results

### Prevalence and characteristics of visually undetectable PCa: training cohort

**Fig. 2:**
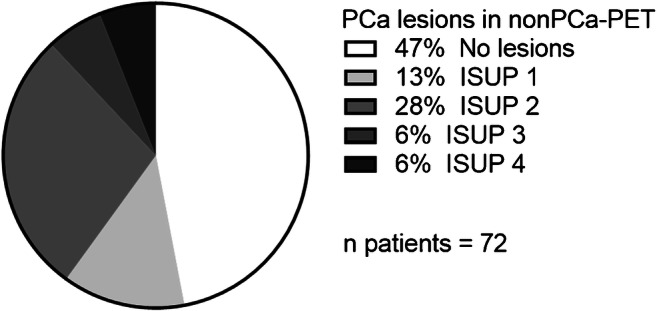
Distribution and ISUP Score of PCa lesions in non-PCa-PET in the entire study cohort

In total, visual PSMA-PET image interpretation missed 134 lesions and the median amount of missed lesions per patient was 2 (range: 0–45 lesions per patient). Visually undetectable lesions were found in 60% (12/20) of the entire prostates and in 45% (18/40) of the prostatic halves, respectively. In 75% of the patients, the visually missed PCa lesions were clinically relevant (ISUP > 1), and in three patients (25%), ISUP ≥ 3 lesions were not detected (Fig. 2 and Supplementary Material 2). The median diameter and the median maximum diameter of the lesions were 2.2 mm (range: 1–6) and 4 mm (range: 2–6), respectively. Most of the lesions had a rounded shape (*n* = 81, 60.4%) and were located in the peripheral zone of the prostate (*n* = 81, 60.4%). Please see Table 2 for a detailed overview of the characteristics of the PCa lesions in non-PCa-PET.

**Table 2:** Characteristics of PCa lesions in non-PCa-PET

			Diameter		Localization (*n* lesions)		Shape (*n* lesions)		
Patient	*n* lesions	Maximum ISUP score	Maximum diameter (mm)	Median diameter (mm)	Peripheral zone	Transitional or central zone	Lenticular	Round	Other
1	29	1	6	2.4	17	12	15	13	1
2	9	2	5	3	7	2	6	2	1
3	0								
4	15	3	4	1.4	7	8	4	11	0
5	2	2	3	3	2	0	2	0	0
6	11	1	3	1.2	8	3	1	10	0
7	2	2	2	1.5	1	1	1	1	0
8	2	2	6	3.5	2	0	0	1	1
9	0								
10	45	1	5	1.4	24	25	10	32	3
11	3	2	5	2.3	2	1	0	2	1
12	1	2	2	2	1	0	1	0	0
13	0								
14	0								
15	0								
16	13	4	4	2.5	10	3	5	7	1
17	2	4	3	2	0	2	0	2	0
18	0								
19	0								
20	0								
Total	134				81	53	45	81	8
Median	2	2	4	2.2	4.5	2	1.5	2	0.5
IQR	(0–10.5)	(1–3)	(3–5)	(1.4–2.9)	(1.3–9.5)	(0.3–6.8)	(0.3–5.8)	(1–10.8)	(0–1)

### Detection of visually undetectable PCa: training cohort

**Fig. 3:**
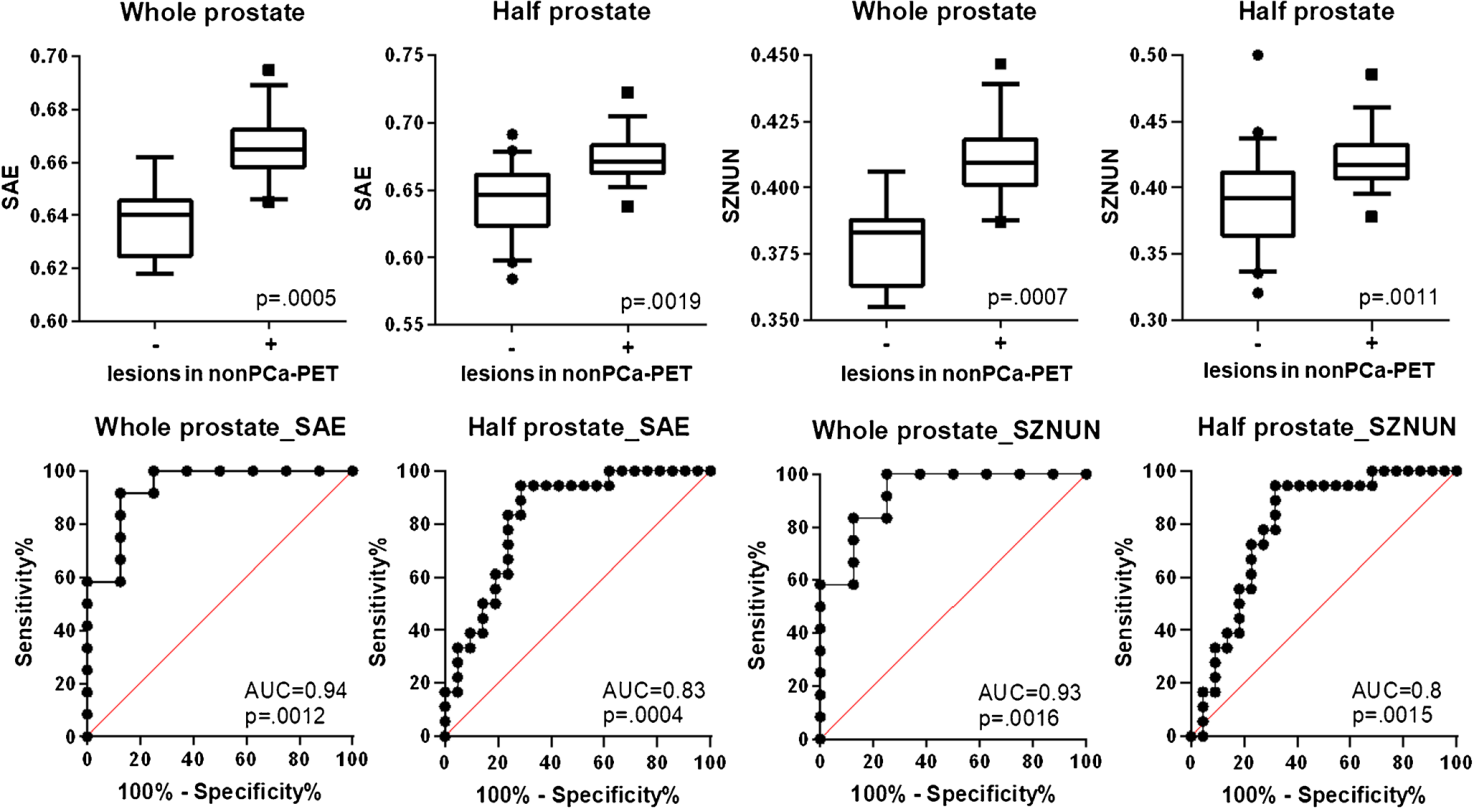
Performance of SAE and SZNUN in the training cohort. Two-tailed Mann-Whitney *U* test including *p* adjustment with Benjamini and Hochberg’s false discovery rate revealed highly significant (*p* < 0.001) differences between whole glands with or without lesions in non-PC-PET for both RFs. The same result was observed on half-gland level. ROC analyses with both RFs resulted in area under the curve (AUC) values of ≥ 0.8 in half prostate level analysis and AUC values ≥ 0.93 in whole prostate level analysis. In the upper row, boxplots are shown: the middle line in the box represents the median and the upper and lower ends of the box represent the 75th and 25th percentiles, respectively. The minimum and maximum values are also shown. In the lower row, ROC-AUC curves are depicted: the black line represents the respective ROC curve and the red line represents the random classification. SAE, local binary pattern small-area emphasis; SZNUN, local binary pattern size-zone non-uniformity normalized

Firstly, a half-gland analysis was performed by dividing non-PCa-PET for each patient into two-halves (left and right lobes). Of the 154 extracted RFs, 152 had no statistically significant differences in non-PCa-PET areas with or without lesions (*p* > 0.05, see Supplementary Material 3). A strong trend (before and after adjusting for multiple testing: *p* = 0.002 and *p* = 0.059) was observed for SUVmax within non-PCa-PET after applying the LBP filter. Two distinct texture features, the LBP size-zone non-uniformity normalized (SZNUN) and LBP small-area emphasis (SAE), were found to be significantly different in non-PCa-PET areas with or without lesions (*p* ≤ 0.01, Fig. 3). ROC analyses revealed AUCs of 0.8 (95% CI: 0.65–0.94) and 0.8 (95% CI: 0.70–0.96) for SZNUN and SAE, respectively. Subsequently, ROC analysis was applied to derive a RF feature threshold yielding a sensitivity of 100% for both RFs (SZNUN = 0.377, SAE = 0.637). By applying the latter thresholds, we calculated positive and negative predictive values for SAE and SZNUN of 0.74 and 0.85, respectively. Spearman’s test revealed a strong correlation (*r* = 0.85, *p* < 0.001) between both RFs in half-gland analysis. Both RFs had a moderate correlation with the volume of the respective non-PCa-PET area: SZNUN (*r* = 0.26, *p* = 0.1) and SAE (*r* = 0.42, *p* = 0.04).

Secondly, whole prostate analysis was performed by assessing the impact of basic clinical parameters on the distribution of visually undetected PCa lesions. For the following clinical parameters, no significant differences were found for patients with or without PCa lesion in non-PCa-PET: NCCN risk group (*p* = 0.642), iPSA (*p* = 0.326), ISUP score of the primary lesion (*p* = 0.146), pT stage (0.256), SUVmean in GTV-PET (*p* = 0.691), and SUVmax in GTV-PET (*p* = 0.658). For the two RFs SZNUN and SAE, ROC analysis revealed AUCs of 0.93 (95% CI: 0.81–1) and 0.94 (95% CI: 0.83–1), respectively (Fig. 3). Additionally, RF feature threshold yielding a sensitivity of 100% was calculated for both RFs (SZNUN = 0.386, SAE = 0.645) by ROC analyses. Applying the latter thresholds, the resulting positive and negative predictive values were 0.86 and 0.75 for SAE and for SZNUN 0.85 and 0.75, respectively. In Spearman’s test, a very strong correlation (*r* = 0.99, *p* < 0.001) between the two RFs was observed. Both RFs had a weak correlation with the volume of non-PCa-PET: SZNUN (*r* = 0.37, *p* = 0.1) and SAE (*r* = 0.4, *p* = 0.08).

### Prevalence and detection of visually undetectable PCa: validation cohort

Visual PET image interpretation missed at least one PCa lesion in 50% (26/52) of the patients in the external validation cohort. Of these patients, 77% (20/26) possessed clinically significant but visually undetected PCa (Fig. 2 and Supplementary Material 2). For non-resampled images in whole-gland analysis, sensitivities of 85% for SZNUN (cut point: 0.386) and 81% for SAE (cut point: 0.645), respectively, were observed. After resampling, the sensitivity decreased to 23% for SZNUN (cut point: 0.386) and 19% for SAE (cut point: 0.645), respectively.

### ISUP grade characterization

**Fig. 4:**
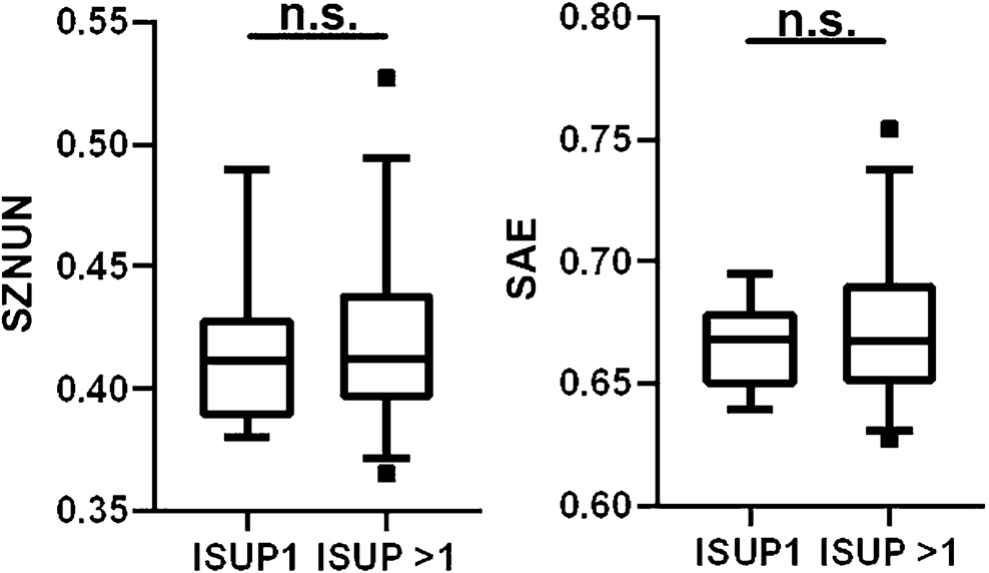
Performance of RF SAE and SZNUN for ISUP1 and ISUP > 1 discrimination. Two-tailed Mann-Whitney *U* test revealed no significant (*p* > 0.05) differences for both RFs between non-visible lesions with different ISUP scores. Boxplots are shown: the middle line in the box represents the median and the upper and lower ends of the box represent the 75th and 25th percentiles, respectively. The minimum and maximum values are also shown. SAE, local binary pattern small-area emphasis; SZNUN, local binary pattern size-zone non-uniformality normalized

Finally, patients of both cohorts with PCa in non-PCa-PET were pooled for further analyses. Both RFs failed to discriminate between patients with ISUP1 and ISUP > 1 PCa in non-PCa-PET (SAE: *p* = 0.8, SZNUN: *p* = 0.79, Fig. 4).

## Discussion

PSMA-PET/CT was introduced as an image modality with a high sensitivity (64–89%) for intraprostatic tumor detection [15, 36] and the first studies are implementing PSMA-PET information to guide a focal RT [37] by increasing the RT dose to the visible intraprostatic tumor mass. Most possibly, an ultra-focal treatment targeting solely the visible tumor mass in PSMA-PET images would reduce the treatment-related side effects due to better protection of the adjacent organs like rectum and bladder. However, treatment outcomes after ultra-focal therapies targeting solely the visible tumor mass in PSMA-PET images have not been reported yet. One main obstacle to this approach is that intraprostatic tumor lesions may be missed by visual PSMA-PET image interpretation due to two main reasons. First, detection of small lesions and of lesions with lenticular shape is hampered by the physical limitations of PET scanning systems as it was described for choline-PET previously [18]. Second, intra- and intertumoral differences in PSMA expression were reported and approximately 10% of all intraprostatic lesions have no PSMA expression [38]. Consequently, appropriate patient selection remains demanding for ultra-focal therapy approaches. To the best of our knowledge, this is the first report to examine the amount and the characteristics of the visually undetected intraprostatic tumor in PSMA-PET images. Additionally, we present two distinct RFs for their discrimination.

Taken together, 53% of the patients in our study had visually not detected PCa and 40% possessed clinically significant PCa with ISUP > 1. The visually undetected lesions were mainly small (median diameter 2.2 mm) and approximately one-third had a lenticular shape. This observation is in accordance with the study of Souvatzoglou et al. [18]. The majority of the lesions (60.4%) were located in the peripheral zone which is also in concordance with previous studies [17]. The invisible lesions were equally distributed in patients with differences in basic clinical parameters (like iPSA, pT stage, and NCCN risk group) and basic SUV parameters derived from GTV-PET (SUVmean and SUVmax). Consequently, other markers are warranted to predict the presence of invisible but clinically significant PCa lesion. Our group investigated RF from non-PCa-PET for the prediction of multifocality of PCa as a biomarker. Basic PET parameters from non-PCa-PET like SUVmean and SUVmax failed to discriminate patients with invisible lesions, although a strong trend for SUVmax was observed. However, two texture features (SAE and SZNUN) showed excellent performance, with ROC-AUC of ≥ 0.8–0.94 in the detection of invisible PCa lesions. The satisfying performance was repeatable in the external validation cohort with calculated sensitivities of ≥ 80% for non-resampled PET images. These findings may be explained by the technical properties of both RFs: quantification of the amount and distribution of small areas with PSMA uptake within the area of interest. The LBP filter was applied to enhance the detection of small areas with tracer uptake to notice even small PCa lesions. The LBP filter was already successfully applied by Kwak and colleagues [39] in a computer-aided diagnosis system for the detection of prostate cancer, and on other malignant diseases as well [34]. After resampling, the sensitivity decreased, suggesting that both RFs are very sensitive to image processing. Additionally, the spatial resolution in the validation cohort (2.3 × 2.3 × 2.7 mm) is lower than the median diameter of the missed lesions (2.2 mm) and subsampling the data to 2 × 2 × 2 mm will probably not help to reconstruct smaller lesions.

A reliable prediction of the presence of visually non-detectable but clinically significant PC might have impact on future therapeutic approaches and support decision-making. In patients with a high risk for clinically significant PCa in non-PCa-PET, ultra-focal therapy approaches targeting solely the visible tumor mass should be omitted and treatments targeting the entire prostatic gland (e.g., prostatectomy or conventional RT) should be offered. By applying predefined thresholds for both RFs, we calculated positive predictive values of > 0.85 in whole-gland analysis which indicates that the chance of falsely omitting focal treatments is low. Interestingly, we could reproduce the strong performance of both RFs from whole-gland analyses also by considering half of the prostatic gland in our training cohort. Prostatic halves with low risk for the presence of non-visible PCa lesions in PET may be spared from therapy (e.g., half-gland therapy) which may result in a significant reduction of treatment-related side effects [5]. Future studies should also address the implementation of RF-based detection of PCa lesions in clinical routine workflows. First, an accurate segmentation of the visible tumor mass in PSMA-PET and mpMRI and the prostatic gland in CT or mpMRI should be performed manually or by using automatic tools (for example deep learning approaches [40, 41]). Second, RF should be extracted by implementing already developed software tools for RF calculation [42]. The entire workflow should be integrated into the standard software solutions for focal therapy or for targeted biopsy guidance. It should be mentioned that in 10–20% of the patients PCa lesions were missed by visual PET image interpretation and by implementation of RFs. The abovementioned lack of PSMA expression in approximately 10% of the PCa lesions may serve as an explanation for this result. In a study by Touijer et al. [43], immunohistochemical analyses revealed that gastrin-releasing peptide receptor (GRPR) expression is not correlated with PSMA expression, suggesting that PET imaging targeting the GRPR may offer complementary information to PSMA-PET imaging. Thus, future studies should also assess the RF extraction from other imaging modalities in order to decrease the chance of missing lesions. It should be mentioned that our approach does not provide any information on the localization of the visually non-detected lesions. McGarry et al. [44] implemented PCa radiomics to generate probability maps that generate a probability of malignancy in MR images on a voxel level. Whether this approach is applicable in PSMA-PET images should be addressed in further studies.

Sowalsky et al. [45] postulated that a subset of PCa lesions with Gleason patterns 3 and 4 have a common origin. This finding supports a branched evolution model wherein Gleason pattern 3 and 4 tumors emerge from a common precursor. Additionally, Haffner et al. [46] proposed that lesions with ISUP1 could be lethal for PCa patients as well. In contrary, recurrence-free survival after surgery or RT is significantly increased in patients with ISUP 1 [47]. Consequently, other studies defined PCa lesions with ISUP > 1 as clinically relevant [25]. Bravaccini et al. [48] advocated that PSMA expression on PCa cells correlates with the ISUP score. However, in our study, both RFs failed to discriminate whether the lesions possessed clinically relevant PCa in terms of ISUP. This might be explained by the small size of the lesions in non-PCa-PET which probably results in minimal differences in measurable PET signal. Thus, differences in PSMA expression due to various ISUP scores will not result in significant diversity in PET signal and consequentially in RFs. Nevertheless, we suggest that both RFs deliver clinically relevant information despite the missing discrimination between ISUP 1 and ISUP > 1 lesions, since both RFs detected prostates with invisible PCa in visual PET analysis which revealed clinically significant PCa in the vast majority. Future studies integrating genomic information despite solely the Gleason score should re-analyze whether invisible but clinically significant PCa lesion with ISUP1 may be characterized with RF. A few papers on radiogenomics have been published already [49, 50].

Due to an elaborate but labor-intensive PET histopathology registration protocol with high spatial resolution, a limitation of our study is the low number of patients in the training cohort. Considering the low number of patients and the non-significant effect of the standard clinical parameters (like PSA and NCCN risk group) on the distribution of the visually undetectable lesions, no additional multivariate regression analysis was performed in order to exclude confounding variables. In the validation cohort, only patients with PCa in non-PCa-PET were considered for RF analyses because of two reasons. Due to the relatively low resolution of histopathology information (5 mm between whole-mount slices) in the validation cohort, the chance of missing lesions in non-PCa-PET (approx. 1–5 mm diameter) was not negligible. Thus, it could not be excluded that false-negative findings of RF were due to the missed lesions in histopathology information instead of poor RF performance. Additionally, no dedicated co-registration protocol between histopathology slices and PET images was applied in this cohort. Likewise, a clear differentiation between small branches of the main tumor mass and autonomous lesions close to the main tumor mass was not always possible. In this study, the PSMA expression on the whole-mount tissue slices was not assessed by immunohistochemistry. Thus, it remains unclear how many of the visually undetectable lesions had no PSMA expression. Although MRI is the actual SOC for primary PCa staging, we decided to extract RFs from PSMA-PET images, as a higher sensitivity to detect primary PCa lesions has been reported for PSMA-PET when compared to MRI. The tracer that was injected in this study was ^68^Ga-PSMA-11. It is not clear whether the results of our study can be translated for ^18^F-labeled tracers. A recent study by Kuten et al. [51] observed comparable results in PCa detection between ^68^Ga-PSMA-11 and ^18^F-PSMA-1007. However, ^18^F-PSMA-1007 seems to detect additional lesions of limited clinical relevance [51]. The impact of PET-tracer on RFs is being controversially discussed [52]. In this study, no multiple delineations of GTV-PET were obtained. Thus, it remains unclear how sensitive both RFs are to the quality of initial GTV-PET (and consequently non-PCa-PET) segmentation. However, in this work, we used a validated contouring approach for manual PET image segmentation and previous work revealed a very good interobserver agreement when using this technique [23]. Additionally, both RFs performed well in whole- and half-gland analyses. This underlines the robustness of both RFs for invisible lesion discrimination independent of the analyzed volume of interest. Considering the limitations of our study, future work implementing histopathology reference with higher resolution should further validate the ability of RFs to detect primarily not visible but clinically significant PCa. We emphasize that RFs cut point values derived from our patient cohorts have been provided as a proof of concept and the optimal thresholds should be further investigated.

To conclude, visual ^68^Ga-PSMA-11 PET image interpretation missed small but clinically significant PCa in a relevant number of patients. However, two distinct RFs provided a very good performance in their detection. This may improve personalized diagnostic and therapeutic approaches for primary PCa by providing complementary information to visual PET interpretation.

## Supplementary information

ESM 1 (DOCX 33 kb).

## Data Availability

The authors confirm that the data supporting the findings of this study are available within the article (and/or) its supplementary materials.

## Abbreviations

GTV: gross tumor volume
ISUP: International Society of Urological Pathology Score
LBP: local binary pattern filter
mpMRI: multiparametric magnetic resonance imaging
non-PCa-PET: non-prostate cancer tissue in visual PSMA-PET image interpretation
RFs: radiomics features
RT: radiotherapy
SOC: standard of care
NCCN: National Comprehensive Cancer Network
PCa: prostate cancer
PSMA-PET: prostate-specific membrane antigen positron emission tomography
ROC-AUC: area under the receiver-operating characteristic curve (AUC-ROC)
SAE: small-area emphasis
SUV: standardized uptake value
SZNUN: size-zone non-uniformity normalized
