# Supplementary material for: Uncovering the invisible—prevalence, characteristics, and radiomics feature–based detection of visually undetectable intraprostatic tumor lesions in 68GaPSMA-11 PET images of patients with primary prostate cancer
Source: Eur J Nucl Med Mol Imaging. 2020 Nov 18;48(6):1987–97. doi: 10.1007/s00259-020-05111-3 (PMC8113179; doi:10.1007/s00259-020-05111-3)
Supplement: Supplementary file 1 — (DOCX 33 kb). [file 259_2020_5111_MOESM1_ESM.docx]

**Supplementary material 1. Radiomic Feature Extraction**

A feature reduction (initial number of features 237) was performed upfront by a phantom study to discard RF with significant inter-scanner variability comparing the three different PET/CT scanner systems in Freiburg (BB, TF, V) (20). The NEMA NU2 phantom was employed and 9 contours of variable size and intensity were segmented. In each patient, 154 RF which proofed robust were extracted per volume (entire nonPCa-PET, left lobe and right lobe nonPCa-PET).

Radiomic feature extraction was performed in the original image and by applying a local binary pattern (LBP) filter.

RF were extracted with PyRadiomics version 2.02 (Python 2.07) and settings were defined as follows:

| Settings | padDistance | 10 |
| --- | --- | --- |
|  | preCrop | True |
|  | minimumROIDimension | 3 |
|  | minimumROIsize | 100 |
|  | binWidth | 0.05 |
|  | voxelArrayShift | 0 |
|  | symmetricalGLCM | True |
|  | distances | 1 |
|  | verbose | False |
|  | additionalInfo | True |
|  | weightingNorm | None |
|  | force2D | False |
|  | force2Ddimension | 0 |
|  | interpolator | None |
|  | resampledPixelSpacing | None |
|  | Label | 1 |
|  | normalizeScale | None |
|  | normalize | False |
|  | geometryTolerance | None |
|  | removeOutliers | None |
|  | resamplePixelSpacing | None |
|  | correctMask | False |
|  | resegmentRange | None |
| Filter | Original |  |
|  | LDP3D  (2,3) | customArgs: lbp3DLevels=2, lbp3DIcosphereRadius=1, ldp3DIcosphereSubdivision=1 |
| Feature Classes | First Order Features | |
|  | Gray Level Co-occurrence Matrix (GLCM) Features | |
|  | Gray Level Run Length Matrix (GLRLM) Features | |
|  | Gray Level Size Zone Matrix (GLSZM) | |
|  | Neighboring Gray Tone Difference Matrix (NGTDM) Features | |

**Supplementary material 2. ISUP scores**

|  | **LBP-SZNUN** | **LBP-SAE** | **GS** | **ISUP** | **Training** |
| --- | --- | --- | --- | --- | --- |
| 1 | 0.41 | 0.67 | 3+3 (6) | 1 |  |
| 2 | 0.41 | 0.67 | 3+4 (7a) | 2 |  |
| 3 | 0.40 | 0.66 | 4+3 (7b) | 3 |  |
| 4 | 0.42 | 0.68 | 3+4 (7a) | 2 |  |
| 5 | 0.40 | 0.66 | 3+3 (6) | 1 |  |
| 6 | 0.42 | 0.67 | 3+4 (7a) | 2 |  |
| 7 | 0.40 | 0.66 | 3+4 (7a) | 2 |  |
| 8 | 0.45 | 0.69 | 3+3 (6) | 1 |  |
| 9 | 0.39 | 0.65 | 3+4 (7a) | 2 |  |
| 10 | 0.41 | 0.67 | 3+4 (7a) | 2 |  |
| 11 | 0.41 | 0.66 | 4+4 (8) | 4 |  |
| 12 | 0.39 | 0.65 | 4+4 (8) | 4 |  |
| 1 | 0.41 | 0.67 | 3+4 (7a) | 2 | **External validation** |
| 2 | 0.39 | 0.65 | 3+3 (6) | 1 |  |
| 3 | 0.42 | 0.67 | 3+3 (6) | 1 |  |
| 4 | 0.38 | 0.64 | 4+4 (8) | 4 |  |
| 5 | 0.41 | 0.66 | 3+4 (7a) | 2 |  |
| 6 | 0.40 | 0.66 | 4+3 (7b) | 3 |  |
| 7 | 0.39 | 0.65 | 3+4 (7a) | 2 |  |
| 8 | 0.42 | 0.67 | 3+4 (7a) | 2 |  |
| 9 | 0.38 | 0.64 | 3+3 (6) | 1 |  |
| 10 | 0.41 | 0.67 | 3+4 (7a) | 2 |  |
| 11 | 0.44 | 0.69 | 4+4 (8) | 4 |  |
| 12 | 0.37 | 0.63 | 3+4 (7a) | 2 |  |
| 13 | 0.48 | 0.72 | 4+4 (8) | 4 |  |
| 14 | 0.43 | 0.68 | 3+3 (6) | 1 |  |
| 15 | 0.44 | 0.69 | 4+3 (7b) | 3 |  |
| 16 | 0.40 | 0.65 | 4+3 (7b) | 3 |  |
| 17 | 0.41 | 0.67 | 3+4 (7a) | 2 |  |
| 18 | 0.38 | 0.64 | 3+3 (6) | 1 |  |
| 19 | 0.47 | 0.71 | 3+4 (7a) | 2 |  |
| 20 | 0.49 | 0.73 | 3+3 (6) | 1 |  |
| 21 | 0.42 | 0.68 | 3+4 (7a) | 2 |  |
| 22 | 0.53 | 0.75 | 3+4 (7a) | 2 |  |
| 23 | 0.47 | 0.72 | 3+4 (7a) | 2 |  |
| 24 | 0.46 | 0.71 | 3+4 (7a) | 2 |  |
| 25 | 0.47 | 0.72 | 3+4 (7a) | 2 |  |
| 26 | 0.37 | 0.63 | 4+3 (7b) | 3 |  |

Abbrevations: GS: Gleason Score; LBP-SZNUN: Locally binary pattern-SizeZoneNonUniformityNormalized; LBP-SAE: Locally binary pattern-SmallAreaEmphasis

**Supplementary material 3. Results of half gland analysis for all RFs**

| **Radiomic Feature** | **p value (Mann-Whitney-U)** | **p value after multiple testing adjustment** |
| --- | --- | --- |
| original_firstorder_InterquartileRange |  | 0.101294886 |
| original_firstorder_InterquartileRange.1 |  | 0.101294886 |
| original_firstorder_Skewness | 0.459696625 | 0.610286899 |
| original_firstorder_Uniformity | 0.105790272 | 0.208867973 |
| original_firstorder_RobustMeanAbsoluteDeviation | 0.040123299 | 0.101294886 |
| original_firstorder_MeanAbsoluteDeviation | 0.07815754 | 0.174438567 |
| original_firstorder_Minimum | 0.665857366 | 0.782763621 |
| original_firstorder_Range | 0.682897898 | 0.794610576 |
| original_firstorder_Variance | 0.118756148 | 0.223029839 |
| original_firstorder_Kurtosis | 0.008071496 | 0.065421602 |
| original_glcm_JointEntropy | 0.900294223 | 0.943165377 |
| original_glcm_ClusterShade | 0.748746168 | 0.841656276 |
| original_glcm_MaximumProbability | 0.76996396 | 0.859235144 |
| original_glcm_Idmn | 0.07815754 | 0.174438567 |
| original_glcm_JointEnergy | 0.706877434 | 0.812381529 |
| original_glcm_Contrast | 0.029999778 | 0.095578661 |
| original_glcm_DifferenceEntropy | 0.083167933 | 0.182969452 |
| original_glcm_InverseVariance | 0.029999778 | 0.095578661 |
| original_glcm_DifferenceVariance | 0.140437868 | 0.24576627 |
| original_glcm_Idm | 0.043053704 | 0.105242389 |
| original_glcm_SumSquares | 0.118756148 | 0.223029839 |
| original_glcm_ClusterProminence | 0.257122028 | 0.373554645 |
| original_glcm_DifferenceAverage | 0.034755877 | 0.095578661 |
| original_glcm_Id | 0.043053704 | 0.105242389 |
| original_glcm_ClusterTendency | 0.156460944 | 0.267722059 |
| original_glrlm_GrayLevelVariance | 0.125678623 | 0.230410809 |
| original_glrlm_GrayLevelNonUniformityNormalized | 0.132903555 | 0.237990087 |
| original_glrlm_RunVariance | 0.034755877 | 0.095578661 |
| original_glrlm_GrayLevelNonUniformity | 0.099732281 | 0.204783617 |
| original_glrlm_LongRunEmphasis | 0.017414135 | 0.086508929 |
| original_glrlm_ShortRunHighGrayLevelEmphasis | 0.294401477 | 0.412162067 |
| original_glrlm_RunLengthNonUniformity | 0.212593209 | 0.324152022 |
| original_glrlm_ShortRunEmphasis | 0.010524607 | 0.070469106 |
| original_glrlm_RunPercentage | 0.012497574 | 0.076985056 |
| original_glrlm_LongRunLowGrayLevelEmphasis | 0.834574041 | 0.905101425 |
| original_glrlm_RunLengthNonUniformityNormalized | 0.010524607 | 0.070469106 |
| original_glszm_ZoneVariance | 0.037359707 | 0.100936753 |
| original_glszm_GrayLevelNonUniformityNormalized | 0.14828817 | 0.256588519 |
| original_glszm_SizeZoneNonUniformityNormalized | 0.034755877 | 0.095578661 |
| original_glszm_SizeZoneNonUniformity | 0.530415571 | 0.669540967 |
| original_glszm_GrayLevelNonUniformity | 0.182974117 | 0.293520979 |
| original_glszm_LargeAreaEmphasis | 0.02580119 | 0.095578661 |
| original_glszm_SmallAreaHighGrayLevelEmphasis | 0.164962299 | 0.276132544 |
| original_glszm_ZonePercentage | 0.013598289 | 0.07756061 |
| original_glszm_LargeAreaLowGrayLevelEmphasis | 0.349349747 | 0.476104965 |
| original_glszm_HighGrayLevelZoneEmphasis | 0.257122028 | 0.373554645 |
| original_glszm_SmallAreaEmphasis | 0.032304824 | 0.095578661 |
| original_glszm_ZoneEntropy | 0.173798182 | 0.281736 |
| original_glszm_SmallAreaLowGrayLevelEmphasis | 0.944510102 | 0.963275203 |
| original_ngtdm_Coarseness | 0.307576494 | 0.422917679 |
| original_ngtdm_Complexity | 0.192495415 | 0.302492795 |
| original_ngtdm_Strength | 0.512244487 | 0.657380425 |
| original_ngtdm_Contrast | 0.013598289 | 0.07756061 |
| original_ngtdm_Busyness | 0.442859708 | 0.593046914 |
| lbp.3D.m1_firstorder_Skewness | 0.0604645 | 0.143254353 |
| lbp.3D.m1_firstorder_Median | 0.720028659 | 0.821366026 |
| lbp.3D.m1_firstorder_Energy | 0.173798182 | 0.281736 |
| lbp.3D.m1_firstorder_TotalEnergy | 0.173798182 | 0.281736 |
| lbp.3D.m1_firstorder_Maximum | 0.002123493 | 0.059207189 |
| lbp.3D.m1_firstorder_RootMeanSquared | 0.140437868 | 0.24576627 |
| lbp.3D.m1_firstorder_90Percentile | 0.005860218 | 0.062955852 |
| lbp.3D.m1_firstorder_Minimum | 0.03360906 | 0.095578661 |
| lbp.3D.m1_firstorder_Range | 0.004126357 | 0.059207189 |
| lbp.3D.m1_firstorder_Mean | 0.812893079 | 0.900615354 |
| lbp.3D.m1_glcm_JointAverage | 0.032304824 | 0.095578661 |
| lbp.3D.m1_glcm_SumAverage | 0.032304824 | 0.095578661 |
| lbp.3D.m1_glcm_ClusterShade | 0.125678623 | 0.230410809 |
| lbp.3D.m1_glcm_Autocorrelation | 0.02580119 | 0.095578661 |
| lbp.3D.m1_glcm_Imc2 | 0.900294223 | 0.943165377 |
| lbp.3D.m1_glcm_Imc1 | 0.281601345 | 0.397858781 |
| lbp.3D.m1_glrlm_ShortRunLowGrayLevelEmphasis | 0.922372849 | 0.959766343 |
| lbp.3D.m1_glrlm_LowGrayLevelRunEmphasis | 0.944510102 | 0.963275203 |
| lbp.3D.m1_glrlm_ShortRunHighGrayLevelEmphasis | 0.006132064 | 0.062955852 |
| lbp.3D.m1_glrlm_LongRunLowGrayLevelEmphasis | 0.988893627 | 0.995356984 |
| lbp.3D.m1_glrlm_HighGrayLevelRunEmphasis | 0.011474522 | 0.073628181 |
| lbp.3D.m1_glszm_GrayLevelVariance | 0.002807433 | 0.059207189 |
| lbp.3D.m1_glszm_GrayLevelNonUniformityNormalized | 0.004186791 | 0.059207189 |
| **lbp.3D.m1_glszm_SizeZoneNonUniformityNormalized** | **0.000216046** | **0.001137654** |
| lbp.3D.m1_glszm_SmallAreaHighGrayLevelEmphasis | 0.006727476 | 0.064751954 |
| lbp.3D.m1_glszm_LargeAreaLowGrayLevelEmphasis | 0.032304824 | 0.095578661 |
| lbp.3D.m1_glszm_HighGrayLevelZoneEmphasis | 0.010524607 | 0.070469106 |
| **lbp.3D.m1_glszm_SmallAreaEmphasis** | **0.000246449** | **0.001897653** |
| lbp.3D.m1_glszm_LowGrayLevelZoneEmphasis | 0.548900624 | 0.687241432 |
| lbp.3D.m1_glszm_ZoneEntropy | 0.040123299 | 0.101294886 |
| lbp.3D.m1_glszm_SmallAreaLowGrayLevelEmphasis | 0.567690681 | 0.693844166 |
| lbp.3D.m1_ngtdm_Strength | 0.665857366 | 0.782763621 |
| lbp.3D.m1_ngtdm_Busyness | 0.686254589 | 0.794610576 |
| lbp.3D.m2_firstorder_InterquartileRange | 0.105790272 | 0.208867973 |
| lbp.3D.m2_firstorder_Skewness | 0.014781331 | 0.081297321 |
| lbp.3D.m2_firstorder_Uniformity | 0.003436358 | 0.059207189 |
| lbp.3D.m2_firstorder_Median | 0.303797891 | 0.421485362 |
| lbp.3D.m2_firstorder_Energy | 0.223178357 | 0.333684146 |
| lbp.3D.m2_firstorder_RobustMeanAbsoluteDeviation | 0.118756148 | 0.223029839 |
| lbp.3D.m2_firstorder_MeanAbsoluteDeviation | 0.018874208 | 0.090832128 |
| lbp.3D.m2_firstorder_TotalEnergy | 0.223178357 | 0.333684146 |
| lbp.3D.m2_firstorder_Maximum | 0.251958317 | 0.373092123 |
| lbp.3D.m2_firstorder_RootMeanSquared | 0.512244487 | 0.657380425 |
| lbp.3D.m2_firstorder_90Percentile | 0.118756148 | 0.223029839 |
| lbp.3D.m2_firstorder_Minimum | 0.027582355 | 0.095578661 |
| lbp.3D.m2_firstorder_Range | 0.131174828 | 0.237657924 |
| lbp.3D.m2_firstorder_Variance | 0.004613547 | 0.059207189 |
| lbp.3D.m2_firstorder_Kurtosis | 0.192495415 | 0.302492795 |
| lbp.3D.m2_firstorder_Mean | 0.966689243 | 0.979408838 |
| lbp.3D.m2_glcm_JointAverage | 0.02389483 | 0.095578661 |
| lbp.3D.m2_glcm_SumAverage | 0.02389483 | 0.095578661 |
| lbp.3D.m2_glcm_ClusterShade | 0.008071496 | 0.065421602 |
| lbp.3D.m2_glcm_Autocorrelation | 0.02389483 | 0.095578661 |
| lbp.3D.m2_glcm_SumEntropy | 0.0160516 | 0.085239533 |
| lbp.3D.m2_glcm_MCC | 0.099732281 | 0.204783617 |
| lbp.3D.m2_glcm_SumSquares | 0.004613547 | 0.059207189 |
| lbp.3D.m2_glcm_ClusterProminence | 0.009643531 | 0.070469106 |
| lbp.3D.m2_glcm_Imc2 | 0.665857366 | 0.782763621 |
| lbp.3D.m2_glcm_Imc1 | 0.625791142 | 0.752904968 |
| lbp.3D.m2_glcm_ClusterTendency | 0.017414135 | 0.086508929 |
| lbp.3D.m2_glrlm_ShortRunLowGrayLevelEmphasis | 0.567690681 | 0.693844166 |
| lbp.3D.m2_glrlm_GrayLevelVariance | 0.004186791 | 0.059207189 |
| lbp.3D.m2_glrlm_LowGrayLevelRunEmphasis | 0.567690681 | 0.693844166 |
| lbp.3D.m2_glrlm_GrayLevelNonUniformityNormalized | 0.003436358 | 0.059207189 |
| lbp.3D.m2_glrlm_ShortRunHighGrayLevelEmphasis | 0.02580119 | 0.095578661 |
| lbp.3D.m2_glrlm_RunLengthNonUniformity | 0.099732281 | 0.204783617 |
| lbp.3D.m2_glrlm_LongRunHighGrayLevelEmphasis | 0.027834054 | 0.095578661 |
| lbp.3D.m2_glrlm_LongRunLowGrayLevelEmphasis | 0.58677601 | 0.711523666 |
| lbp.3D.m2_glrlm_RunEntropy | 0.007372803 | 0.065421602 |
| lbp.3D.m2_glrlm_HighGrayLevelRunEmphasis | 0.02580119 | 0.095578661 |
| lbp.3D.m2_glszm_GrayLevelVariance | 0.00379523 | 0.059207189 |
| lbp.3D.m2_glszm_GrayLevelNonUniformityNormalized | 0.006132064 | 0.062955852 |
| lbp.3D.m2_glszm_GrayLevelNonUniformity | 0.269175271 | 0.387411137 |
| lbp.3D.m2_glszm_SmallAreaHighGrayLevelEmphasis | 0.02580119 | 0.095578661 |
| lbp.3D.m2_glszm_LargeAreaLowGrayLevelEmphasis | 1 | 1 |
| lbp.3D.m2_glszm_LargeAreaHighGrayLevelEmphasis | 0.878290649 | 0.939283055 |
| lbp.3D.m2_glszm_HighGrayLevelZoneEmphasis | 0.029999778 | 0.095578661 |
| lbp.3D.m2_glszm_LowGrayLevelZoneEmphasis | 0.856378567 | 0.922253841 |
| lbp.3D.m2_glszm_ZoneEntropy | 0.07815754 | 0.174438567 |
| lbp.3D.m2_glszm_SmallAreaLowGrayLevelEmphasis | 0.834574041 | 0.905101425 |
| lbp.3D.m2_ngtdm_Coarseness | 0.202366916 | 0.311645051 |
| lbp.3D.m2_ngtdm_Strength | 0.476877511 | 0.627684928 |
| lbp.3D.m2_ngtdm_Busyness | 0.281601345 | 0.397858781 |
| lbp.3D.k_firstorder_Skewness | 0.442859708 | 0.593046914 |
| lbp.3D.k_firstorder_10Percentile | 0.494395888 | 0.645228531 |
| lbp.3D.k_firstorder_Kurtosis | 0.530415571 | 0.669540967 |
| lbp.3D.k_glcm_ClusterShade | 0.064550998 | 0.150618995 |
| lbp.3D.k_glcm_Idmn | 0.900294223 | 0.943165377 |
| lbp.3D.k_glcm_Idn | 0.834574041 | 0.905101425 |
| lbp.3D.k_glcm_Correlation | 0.02043723 | 0.095373739 |
| lbp.3D.k_glcm_MCC | 0.049443989 | 0.118974599 |
| lbp.3D.k_glcm_Imc2 | 0.202366916 | 0.311645051 |
| lbp.3D.k_glcm_Imc1 | 0.099732281 | 0.204783617 |
| lbp.3D.k_glszm_SizeZoneNonUniformityNormalized | 0.032304824 | 0.095578661 |
| lbp.3D.k_glszm_GrayLevelNonUniformity | 0.105790272 | 0.208867973 |
| lbp.3D.k_glszm_LargeAreaHighGrayLevelEmphasis | 0.944510102 | 0.963275203 |
| lbp.3D.k_glszm_SmallAreaEmphasis | 0.032304824 | 0.095578661 |
| lbp.3D.k_glszm_ZoneEntropy | 0.164962299 | 0.276132544 |
| lbp.3D.k_ngtdm_Coarseness | 0.099732281 | 0.204783617 |
| lbp.3D.k_ngtdm_Strength | 0.727712602 | 0.824027505 |
